# Effects of experimental ostracism on cortisol, autonomic regulation, and affective states in adolescents with nonsuicidal self-injury

**DOI:** 10.1186/s13034-026-01099-0

**Published:** 2026-05-22

**Authors:** Andreas Goreis, Annika Lozar, Rosa List, Sofia-Marie Oehlke, Diana Klinger, Bettina Pfeffer, Karin Prillinger, Clarissa Laczkovics, Heidi Zesch, Urs M. Nater, Nadine Skoluda, Peter B. Marschik, Laurence Claes, Paul L. Plener, Oswald D. Kothgassner

**Affiliations:** 1 Department of Child and Adolescent Psychiatry, Medical University of Vienna, Vienna, Austria; 2 Comprehensive Center for Pediatrics (CCP), Medical University of Vienna, Vienna, Austria; 3 Comprehensive Center for Clinical Neuroscience and Mental Health (C3NMH), Medical University of Vienna, Vienna, Austria; 4 Department of Clinical and Health Psychology, Faculty of Psychology, University of Vienna, Vienna, Austria; 5 Department of Child and Adolescent Psychiatry and Psychotherapy, University Hospital Heidelberg, Heidelberg University, German Center for Mental Health (DZPG), Heidelberg, Germany; 6 Interdisciplinary Developmental Neuroscience (iDN), Department of Neurology, Medical University of Graz, Graz, Austria; 7 Center of Neurodevelopmental Disorders (KIND), Department of Women’s and Children’s Health, Centre for Psychiatry Research, Karolinska Institutet & Region Stockholm, Stockholm, Sweden; 8 Child and Adolescent Psychiatry and Psychotherapy, German Center for Child and Adolescent Health (DZKJ), University Medical Center Göttingen, Göttingen, Germany; 9 Clinical Psychology, Faculty of Psychology and Educational Sciences, KU Leuven, Leuven, Belgium; 10 Faculty of Medicine and Health Sciences, University of Antwerp, Antwerp, Belgium; 11 Department of Child and Adolescent Psychiatry and Psychotherapy, University of Ulm, Ulm, Germany

## Abstract

**Background:**

Adolescents who engage in nonsuicidal self-injury (NSSI) show a high sensitivity to ostracism, and its effects may be amplified by dysregulated stress-system function. However, ecologically grounded experimental studies examining multilevel stress responses in adolescent mental health remain scarce. We used a novel, ecologically valid in-person ostracism paradigm to assess subjective, autonomic, and endocrine responses in adolescents with recent NSSI and to characterize stress trajectories.

**Methods:**

Fifty adolescents with recent NSSI (mean age = 16.40 years, 76% female) were randomized (1:1) to live social inclusion or exclusion in a face-to-face ball-toss task based on the Cyberball paradigm. Salivary cortisol was sampled at five points from pre-task to 40 min post-task. Heart rate (HR) and heart rate variability (HRV) were recorded at baseline and during the interaction. Subjective stress, tension, and NSSI urges were assessed before and after the task. Perceived exclusion and psychological need threat were also assessed.

**Results:**

We found clear ostracism effects on perceived exclusion and basic psychological need threat. Cortisol declined across the paradigm in both conditions (inclusion/exclusion), but the decline was flatter after exclusion, indicating attenuated hypothalamic-pituitary-adrenal axis downregulation. HR decreased modestly over time across conditions, and HRV diverged by condition: it decreased during exclusion and increased during inclusion. There were no condition-related changes in self-reported stress, tension, or NSSI urges.

**Conclusions:**

These findings suggest a decoupling between subjective and psychobiological stress systems in adolescents with NSSI in response to ostracism, characterized by rapid parasympathetic withdrawal and a blunted HPA axis downregulation. This pattern may reflect heightened physiological sensitivity to social threat despite limited conscious distress.

*Trial registration*: This study has been registered at the German Clinical Trials Register (ID: DRKS00025905, https://drks.de/search/en/trial/DRKS00025905).

## Background

Nonsuicidal Self-Injury (NSSI) is typically defined as the deliberate destruction of one’s own body tissue without suicidal intent and for purposes that are not socially or culturally sanctioned [1]. NSSI encompasses behaviors such as cutting, scratching, burning, or hitting oneself [2]. The DSM-5 lists NSSI in Section III as a condition proposed for formal diagnosis termed Nonsuicidal Self-Injury Disorder [3, 4]. The criteria include engaging in NSSI on at least five days in the previous 12 months. Among adolescents, NSSI affects 16–22% of individuals globally [5, 6] and its prevalence is increasing (see [7]. Critically, NSSI is often associated with subsequent suicide attempts [8, 9], underscoring its clinical relevance in adolescents.

The reasons why people engage in NSSI vary. NSSI is most commonly used to regulate aversive affect or cope with stress [10, 11]. Contemporary models of NSSI, such as the Four-Function Model [12, 13], distinguish intrapersonal functions (decrease aversive affect or increase desired affect) from interpersonal functions (decrease interpersonal demands, promote help-seeking behavior). Notably, meta-analytic evidence indicates that intrapersonal functions are endorsed more frequently than interpersonal ones [14]. With respect to precipitants of NSSI, colloquially referred to as “triggers,” the literature differentiates interpersonal factors (e.g., rejection, criticism) from intrapersonal factors (heightened negative affect, perceived stress, tension; see [15, 16]). An interplay between these domains is anticipated by current frameworks [12, 13], and converging evidence from qualitative studies [17], cross-sectional research [18], and ambulatory assessment [15, 19] shows that interpersonal events, together with affective distress and difficulties in emotion regulation (i.e., the reinforcing function of NSSI), frequently act as proximal triggers of NSSI.

While the social, behavioral, and psychological aspects of NSSI have gained considerable traction (and evidence-based treatments have been identified, most importantly dialectical behavior therapy; see [20, 21], less research is available on the neurobiology of NSSI [22]. Current evidence highlights social stressors and their neurobiological underpinnings, specifically, the stress systems of the autonomic nervous system (ANS), and the hypothalamic–pituitary–adrenal (HPA axis) as central factors.

The ANS, among other functions, primarily regulates organ function at rest (parasympathetic ANS) and during stress (sympathetic ANS [23]). In people who engage in NSSI (both adolescents and adults), meta-analytic evidence shows that basal parasympathetic ANS activity (i.e., at rest), often operationalized as heart rate variability, is comparatively lower than in healthy controls, with effects persisting even after stressors have subsided [10]. Rather than indicating parasympathetic dysregulation per se, lower resting parasympathetic activity may reflect reduced autonomic regulatory capacity or flexibility (see also [24]), which is noteworthy given that markers such as heart rate variability and respiratory sinus arrhythmia are commonly interpreted as indices of emotion regulation capacity [25, 26]. Regarding the HPA axis, studies typically assess stress trajectories using salivary or serum cortisol [27]. In this regard, meta-analytic findings indicate blunted cortisol responding, characterized by flatter response curves with reduced reactivity and/or recovery, pointing to proximal neuroendocrine alterations associated with NSSI [10].

Adolescence is a developmental period during which social belonging becomes paramount, accompanied by heightened sensitivity to social rejection as a stressor [28, 29]. Peer victimization and, more broadly, ostracism—the deliberate or implicit ignoring or exclusion of an individual by others, characterized by a lack of social acknowledgment or interaction [30]—pose clear risks for adolescent well-being and appear particularly salient among youth with recent NSSI [31–33]. Bullying, as one form of ostracism, can be considered a risk factor for NSSI [34, 35]. Consistent with the Temporal Need-Threat Model [36], being ignored or excluded undermines core social resources (i.e., belonging, self-esteem, control, and meaningful existence) thereby threatening identity and self-regulation capacities. Converging evidence further indicates that adolescents who engage in NSSI experience more interpersonal stress and aversive life events than peers without NSSI. They also show heightened rejection sensitivity, both in self-report [31] and in neuroimaging studies [37, 38].

Neuroimaging studies show greater activation in medial and ventrolateral prefrontal brain regions—areas implicated in processing social exclusion and in emotion regulation—in rejection contexts [39, 40]. These neural findings align with the neurovisceral integration model [41], which links prefrontal regulatory networks to autonomic control through vagal pathways, with cardiac vagal control commonly indexed by vagally mediated heart rate variability [42]. Under social threat or in response to social stressors, reductions in vagal tone (i.e., lower heart rate variability) may be associated with disrupted autonomic balance, and this pattern may weaken top-down prefrontal emotion regulation capacities and increase vulnerability to NSSI urges and acts in individuals with a history of NSSI (see, e.g. [43]).

Most experimental work on responses to ostracism in NSSI has been conducted in the laboratory using controlled social stressors. The predominant tool is the Cyberball paradigm [30], a computerized virtual ball-tossing game in which participants believe they are playing with two others (actually software agents). Inclusion is modeled by evenly distributed throws, whereas ostracism is induced when the two “co-players” toss only to each other, excluding the participant. Across these studies, affective and perceived-stress outcomes are frequently assessed, yet physiological indices have been collected only sparsely (e.g. [31, 44]). Critically, to our knowledge no prior study has examined whether experimentally induced ostracism elicits NSSI urges in adolescents with recent NSSI or whether such experiences are associated with concurrent HPA axis and autonomic nervous system responses in this population. Beyond these measurement limitations, the traditional computerized format has also been criticized for limited realism and ecological validity [45]. In response, a small number of studies have explored alternative paradigms, such as simulated chat interactions [31] or social-media evaluations [38], but these approaches still lack direct, face-to-face interaction with human partners.

The present study addressed these gaps by experimentally manipulating social inclusion versus exclusion in a randomized between-subjects design, using an ostracism paradigm based on a live, face-to-face Cyberball game [30]: a three-player ball-toss task in which two confederates included or excluded the participant according to a scripted sequence. We measured salivary cortisol at five time points to index HPA axis reactivity to induced ostracism. Before, during, and after the task, we assessed autonomic activity (heart rate and heart rate variability indexed by the root mean square of successive differences [RMSSD]) as well as subjective stress, tension, and NSSI urges. By characterizing multilevel stress responses to social exclusion, our study aims to inform the identification of physiological vulnerability markers and to advance understanding of stress and emotion regulation processes in adolescents with NSSI. Given prior evidence of altered stress responding in NSSI [10], we did not assume a direct correspondence between subjective distress and physiological stress indices and therefore examined these systems in parallel. Our hypotheses were:

1. Excluded adolescents differ from included adolescents regarding their HPA axis response (i.e., lower salivary cortisol).
2. Excluded adolescents differ from included adolescents regarding their ANS response (i.e., higher heart rate and lower heart rate variability).
3. Excluded adolescents differ from included adolescents regarding their subjective response (i.e., higher perceived stress, tension, NSSI urges).

## Method

This study was performed in accordance with the Declaration of Helsinki, and was part of a larger project (“Triggers Online Resulting in Non-Suicidal Self-Injury; TORN”) which was registered in the German Clinical Trials Register (DRKS00025905) and received approval from the Ethics Committee of the Medical University of Vienna (protocol no. 1651/2019).

### Participants

Participants (aged 14–18 years) who fulfilled the DSM-5 NSSI disorder frequency criteria (i.e., self-harm on five or more separate days in the past year) were recruited for this study. We recruited primarily via the in- and outpatient services of the Department of Child and Adolescent Psychiatry at the Medical University of Vienna, Austria, and additionally through social‐media advertisements and referrals from external psychological and psychiatric practitioners. Exclusion criteria were: presence of autism spectrum disorder; severe medical or psychiatric conditions requiring acute treatment (e.g., psychosis); pronounced aggression, or acute suicidality; and no proficient German speaking skills. As per our trial registration (German Clinical Trials Register: DRKS00025905), we aimed for a target sample size of *N* = 50 (*n* = 25 per condition: inclusion vs. exclusion) and ceased recruitment upon reaching this target. An a priori power analysis (G*Power [46]), indicated that *n* = 25 per group provides 80% power to detect a medium between-group effect (*d* ≈ 0.57) at the post-task assessment. This effect size was chosen as a benchmark for effects that are likely to be of clinical and theoretical relevance in stress responding. From a theoretical perspective, ostracism effects are expected to be large (Cohen’s d > 0.8; [47]) for manipulation checks such as perceived exclusion and basic psychological need threat, whereas physiological effects, particularly HPA-axis indices, are expected to be smaller or potentially absent because ostracism paradigms are less socially evaluative than established endocrine stressors such as the Trier Social Stress Test [48]. Accordingly, the study was primarily powered to detect medium-sized effects in autonomic and endocrine indices, which constituted the primary outcomes.

### Procedure

**Fig. 1:**
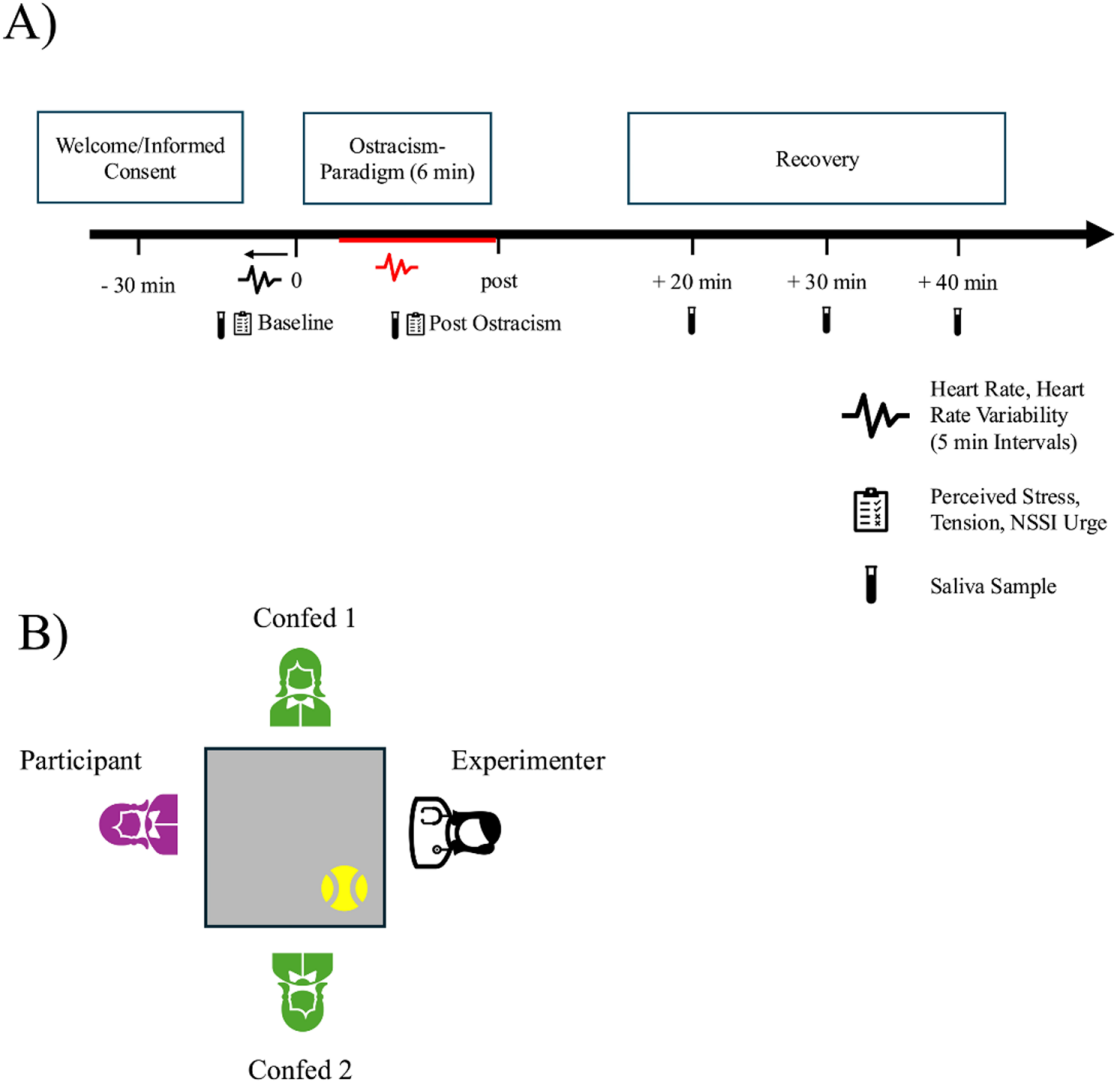
**A** Overview of the study timeline, **B** Schematic of the in-person ball-tossing ostracism paradigm

The study was conducted at the Department of Child and Adolescent Psychiatry, Medical University of Vienna, Austria. Prior to participation, written informed consent was obtained from all adolescents and their legal guardians. Participants were invited to the laboratory in the afternoon. They were instructed to abstain from heavy exercise, alcohol, caffeine, and chewing gum for 24 h prior to the appointment. Furthermore, they were asked not to eat or brush their teeth for one hour before the appointment. After arrival, participants were fitted with heart rate monitors and remained in a quiet laboratory room for 30 min to allow acclimation to the setting and completion of self-report questionnaires. Following the baseline, they were escorted to an adjacent room to complete the 6-minute ostracism paradigm. Subsequently, participants were guided back to the first room, where they completed a series of eye-tracking and cognitive tasks that were part of the larger overarching project and reported elsewhere, see [49] or the trial registration at the German Clinical Trials Register, DRKS00025905, for more details. The first saliva sample (for the determination of salivary cortisol) was obtained directly before the ostracism paradigm, and subsequent saliva samples were taken directly after the paradigm as well as 20, 30, and 40 min later. Overall—including the tasks not reported here—the study took two hours in total. Participants were debriefed after the study and received a €25 voucher for their participation. See Fig. 1A for an overview of the study timeline.

### Ostracism paradigm

Participants completed an in-person ball-tossing ostracism task, conceptually modeled on the Cyberball paradigm [30]. Participants were seated at one side of an 80 × 80 cm wooden table directly across from the experimenter, with two women confederates (undergraduate research assistants whom they believed were fellow participants) seated to the left and right (Fig. 1B). Using a standard tennis ball, all three players were verbally instructed (following a standardized script) to roll the ball across the table to one another as quickly and accurately as possible while the experimenter monitored time with a stopwatch. After an initial one-minute fair-play phase, a covert beep (from the stopwatch) signaled the start of the randomized five-minute condition: in the inclusion condition, the confederates continued to toss the ball equitably; in the exclusion condition, they delivered three more throws to the participant before deliberately excluding them for the remainder of the session. Condition assignment was randomized in advance by a research assistant; participants remained blind to it, while only the two confederates were informed whether to include or exclude the participant (i.e., not tossing the ball to the participant) following a covert signal from the experimenter one minute into the game/experiment. The experimenter was blind to the assigned condition until the signal, after which they could observe how the game unfolded. At the end of the six-minute paradigm, the experimenter escorted the participant to an adjacent room to complete self-report validity checks—estimating the percentage of tosses received, rating perceived ignoring/exclusion during the game, and completing the Reflexive Needs Questionnaire [30].

## Measures

### Reflexive needs questionnaire

The Reflexive Needs Questionnaire [30], a standard manipulation check after ostracism/Cyberball paradigms, assessed participants’ feelings of belonging, self-esteem, control, meaningful existence (five items each) and mood (eight items) during the ostracism task. Participants rated the extent to which each statement reflected their feelings during the game (e.g., “I felt rejected,” “I felt good about myself,” “I felt powerful”; mood items included “I felt good/happy/sad”) on a 5-point Likert scale from 1 (not at all) to 5 (extremely). We computed total and subscale sum scores. The questionnaire was administered immediately after the ostracism task, and the internal consistency of the total score was α = 0.93 in the present study.

### Visual analog scales

To indicate momentary perceived stress, tension, and NSSI urges, participants rated single-item visual analog scales (VAS) by marking a 100-mm line with a pen, anchored at 0 = not at all and 100 = extremely. Ratings were obtained at two time points: before and after the ostracism paradigm (Fig. 1A).

### Nonsuicidal self-injury (NSSI)

We used a short version of the German version of the revised Self-Injurious Thoughts and Behaviors Interview (SITBI-R [50]), a validated, semi-structured interview, to assess NSSI presence, frequency, and characteristics.

### Depressive symptoms

We used the German version [51] of the Beck Depression Inventory-II (BDI-II [52]) to evaluate depressive symptoms experienced in the preceding two weeks. The BDI-II comprises 21 individual questions, each offering four response options on a Likert scale, ranging from 0 to 3. A higher score on a particular item indicates a more pronounced expression of the symptom. The internal consistency of the BDI-II was Cronbach’s α = 0.91 in this sample.

### Perceived stress

Perceived stress was measured with the German version of the Perceived Stress Scale (PSS-10 [53]). The 10 items assess how unpredictable, uncontrollable, and overwhelming daily life felt during the last month, using a 5-point Likert scale (0 = never, 4 = very often) and higher total sum scores denote higher levels of stress. The internal consistency of the scale was α = 0.80.

### Borderline personality disorder (BPD) symptoms

Given the high comorbidity of NSSI with BPD, the Borderline Symptom List-23 (BSL-23 [54]) was administered to screen participants for BPD symptoms. This self-report measure entails 23 items which are to be rated on a 5-point Likert scale (ranging from “not at all” to “very strongly”) and assesses severity of BPD-related symptoms. The internal consistency of the scale was α = 0.89.

### Salivary cortisol

Commercial cotton swabs (Salivette^®^, Sarstedt, Wiener Neudorf, Austria) without any saliva-stimulating additives were used for the determination of salivary cortisol concentrations, a marker of the HPA axis (re)activity. Participants were instructed on how to collect their saliva and to place the swab into their cheek pouch at the designated sampling time points, allowing the swab to saturate with saliva for approximately 120 s. Samples were then stored at -20 °C until analyses were conducted at the biochemical laboratory of the Faculty of Psychology, University of Vienna, Vienna, Austria. The free cortisol concentration (nmol/L) in saliva was determined using a commercial luminescence immunoassay (IBL-Tecan, Hamburg, Germany). Intra- and inter-assay coefficients of variation were below 10% for salivary cortisol.

### Electrophysiological measures

Cardiac signals were continuously recorded with a movisens EcgMove 4^®^ sensor (movisens GmbH, Karlsruhe, Germany; 1024 Hz). The two-electrode sensor was placed below the sternum, and ECG recordings were converted into beat-to-beat heart rate (bpm) and RMSSD (ms) using DataAnalyzer^®^ software (movisens GmbH). For each experimental phase, heart rate and RMSSD were averaged across predefined 5-min segments, in accordance with established heart rate variability guidelines recommending this duration for reliable short-term estimation of RMSSD in experimental settings [55]. Segments were analyzed at baseline (starting 25 min after arrival, directly before the ostracism paradigm) and during the 5-min inclusion/exclusion phase (see Fig. 1A). This segment length was chosen to ensure stable estimation of sustained autonomic activity and consistent comparison across indices. Heart rate was included as a nonspecific marker of cardiac autonomic state, reflecting the relative influence of sympathetic and parasympathetic input on the heart [10], whereas parasympathetic regulation was primarily indexed using heart rate variability as the more informative electrophysiological outcome. Both measures were derived concurrently from the same ECG recording (i.e., the movisens EcgMove 4^®^ sensor).

### Statistical analyses

All analyses were conducted in R (4.4.2) using the packages lme4 for mixed-effects models, lmerTest for *p*-values with Satterthwaite degrees of freedom, emmeans for estimated marginal means and Tukey-adjusted contrasts, and ggplot2 for visualization. For manipulation checks assessed once after the paradigm (perceived exclusion, toss-percentage estimates, Reflexive Needs Questionnaire scores), we used independent-samples t-tests. For repeated-measures outcomes (stress, tension, NSSI urges, heart rate, and heart rate variability), we fit mixed-effects models with fixed effects of group (inclusion vs. exclusion), time (two levels: baseline and during/after ostracism), and their group x time interaction; participant ID was included as a random intercept. Effects are reported with Type III *F*-tests and *α* = 0.05 (two-tailed). Where appropriate, we report Hedges’ *g* for between-group differences (with 0.2/0.5/0.8 denoting small/medium/large effects [56]).

Cortisol trajectories were modeled with orthogonal polynomial time terms across five samples: a linear component (time) indexing overall monotonic change and a quadratic component (time^2) indexing curvature. Fixed effects included group, the linear and quadratic time terms, and group x time interactions for each component; random intercepts were specified for participants. The linear term tests whether salivary cortisol levels show a systematic increase or decline across the assessment period; the quadratic term tests for curvature (e.g., rebound or non-linear recovery). Because salivary cortisol is right-skewed, values were natural log-transformed prior to analysis. Implausible values (< 1 nmol/L or > 60 nmol/L [57]), were excluded (7 values), and 27 values were missing due to insufficient sample volume, likely reflecting low salivary flow, dry mouth, or variability in Salivette swab saturation. No imputation was performed, as mixed-effects models provide unbiased fixed-effect estimates under a missing-at-random assumption, which was supported by Little’s MCAR test, χ²(26) = 14.40, *p* = .968.

## Results

The sample consisted of 50 participants (*M* = 16.40 years, *SD* = 1.33, 76% identified as female, Table 1). Twenty-five of those participants were randomized to the ostracism paradigm inclusion condition and 25 to the exclusion condition. Regarding NSSI, participants reported 1.67 episodes in the past week, 6.40 in the past month, and 114.52 in the past year. The most common methods were cutting oneself (97.9%), scratching (81.3%), punching (70.8%), burning (58.3%), biting (52.1%), and inserting objects under nails or skin (17%). Depressive symptoms (*M* = 32.78, *SD* = 12.94), symptoms of borderline personality disorder (*M* = 50.73, *SD* = 23.19), and perceived stress (*M* = 25.75, *SD* = 7.34) were also elevated according to the respective screening instruments. No group differences (inclusion vs. exclusion) were observed in baseline characteristics (all *t*- and *χ²*-tests: *p*s > .212), indicating successful randomization. Table 1 additionally provides an overview of sample characteristics and ICD-10 diagnoses (total and by condition).

**Table 1:** Participant characteristics

Characteristic	Mean (SD)		
	Total Sample	Inclusion Condition	Exclusion Condition
Age (years)	16.40 (1.33)	16.32 (1.41)	16.48 (1.26)
Age at first NSSI (years)	12.08 (2.25)	12.04 (1.57)	12.13 (2.80)
NSSI 1-week prevalence (SITBI-R)	1.67 (3.75)	2.00 (5.03)	1.33 (1.79)
NSSI 4-week prevalence (SITBI-R)	6.40 (7.49)	6.13 (6.96)	6.67 (8.12)
NSSI 1-year prevalence (SITBI-R)	114.52 (173.57)	85.38 (114.18)	143.67 (216.22)
Perceived Stress Scale-10	25.75 (7.34)	24.80 (6.71)	26.44 (7.78)
Beck Depression Inventory-II	32.78 (12.94)	30.48 (12.35)	35.08 (13.35)
Borderline Symptom List-23	50.73 (23.19)	45.76 (21.06)	53.56 (25.92)
		*n (%)*	
Gender			
Female	38 (76%)	21 (84%)	17 (68%)
Male	3 (6%)	1 (4%)	2 (8%)
Gender-diverse	9 (18%)	3 (12%)	6 (24%)
Previous ICD-10 diagnoses			
F3x Mood (affective) disorder	23 (46%)	13 (52%)	10 (40%)
F4x Neurotic, stress-related and somatoform disorders	23 (46%)	10 (40%)	13 (52%)
F5x Behavioral syndromes associated with physiological disturbances and physical factors	7 (14%)	3 (12%)	4 (16%)
F6x Disorders of personality and behavior	21 (42%)	10 (40%)	11 (44%)
F9x Behavioral and emotional disorders	9 (18%)	6 (24%)	3 (12%)

### Ostracism paradigm responses

**Fig. 2:**
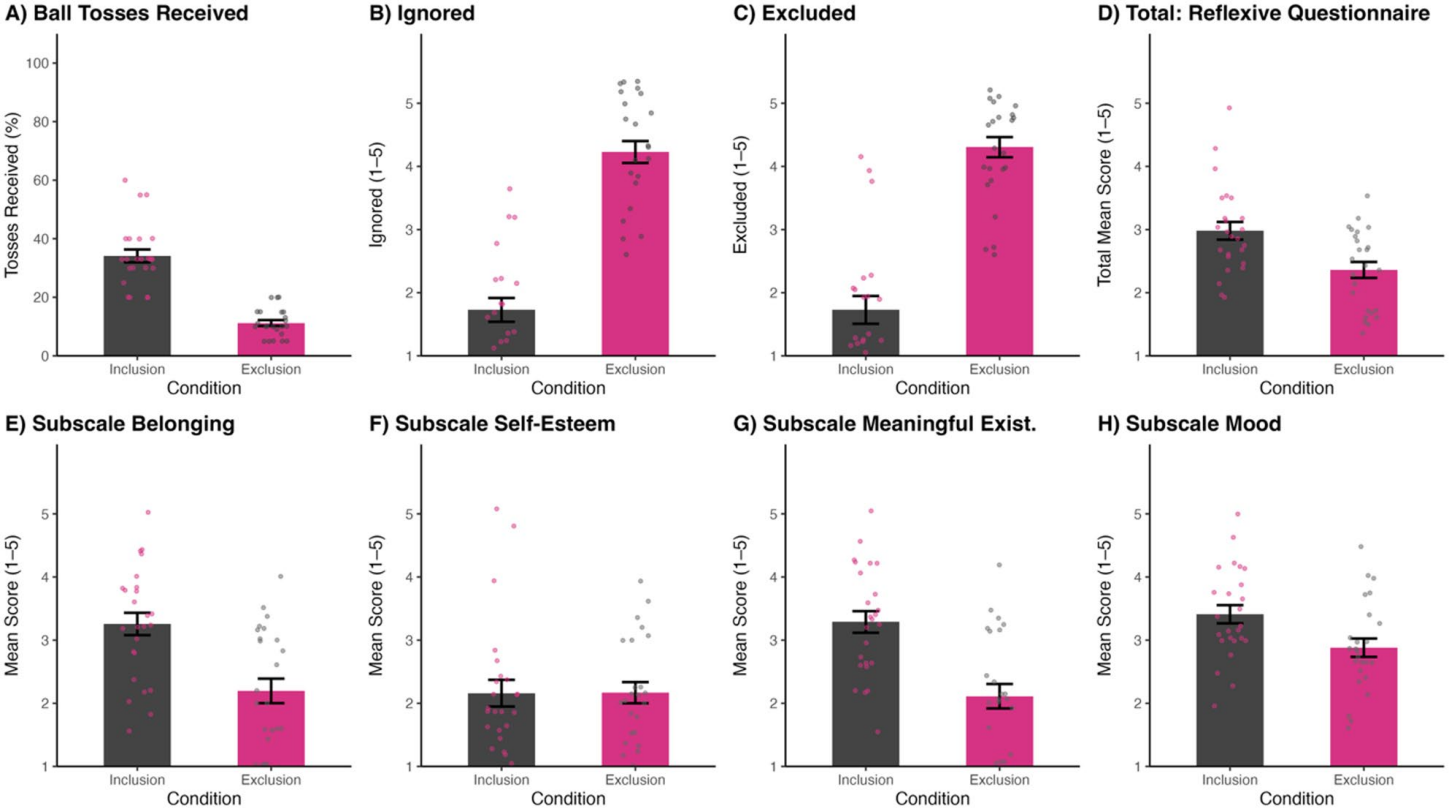
**A–C** In-person ball-tossing ostracism paradigm single-item validation checks, **D** Total mean score of the Reflexive Needs Questionnaire, **E**–**H** Subscales of the Reflexive Needs Questionnaire. Higher values on the Mood subscale indicate more positive mood (i.e., less negative affect following the ostracism task). Error bars represent the standard error of the mean (SEM)

The ostracism task exerted a robust impact on perceived ostracism and basic psychological needs: Participants in the exclusion condition reported receiving substantially fewer tosses than those in inclusion (11.20% vs. 34.13%, *t*(32.28) = 9.53, *p* < .001; Fig. 2A). On single-item ratings, excluded participants felt more ignored (*g* = 2.89, *t*(41.72) = 9.77, *p* < .001; Fig. 2B) and more excluded (*g* = 2.80, *t*(38.67) = 9.48, *p* < .001; Fig. 2C).

The Reflexive Needs Questionnaire total score was lower after exclusion, indicating greater basic need threat (*g* = -0.91, *t*(47.51) = 3.28, *p* = .002; Fig. 2D). At the subscale level, exclusion most strongly reduced meaningful existence (*g* = -1.27, *t*(47.29) = 4.56, *p* < .001), followed by belonging (*g* = -1.12, *t*(47.59) = 4.04, *p* < .001) and mood (*g* = -0.72, *t*(47.00) = 2.59, *p* = .013; Figs. 2E–H). Self-esteem did not differ between conditions (g = -0.03, *t*(45.66) = 0.03, *p* = .976). Together, these results confirm the impact of social exclusion in the paradigm, except for self-esteem.

### Cortisol, autonomic, and affective responses

**Fig. 3:**
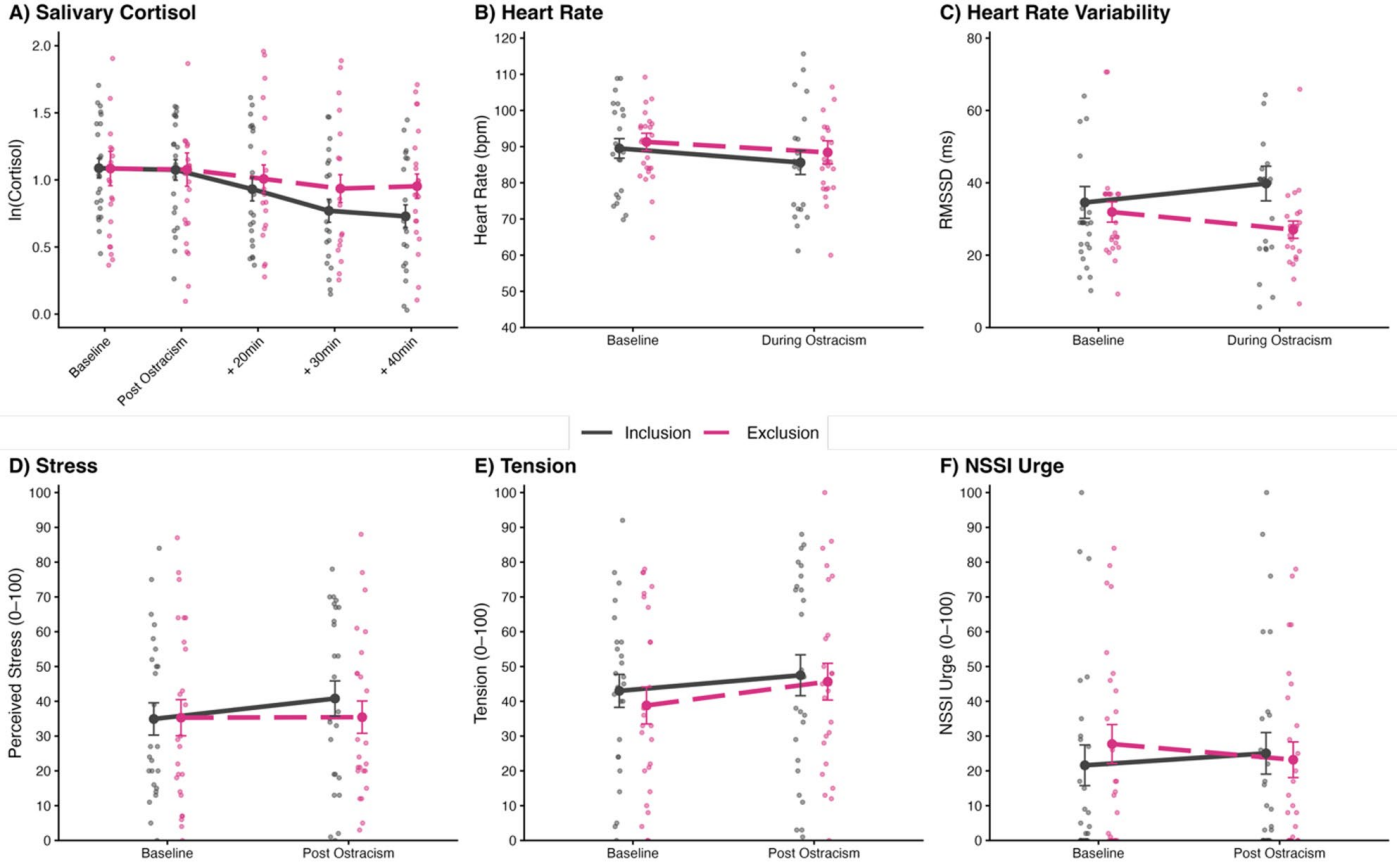
**A** Log Salivary Cortisol values over the course of the paradigm. **B–F** Pre-post differences of heart rate, heart rate variability, subjective stress, tension, and NSSI urge (single item). Error bars represent the SEM

Salivary cortisol (log-transformed, Fig. 3A) showed a robust effect of time: in the orthogonal-polynomial model, the linear time component was strongly negative (*b* = -1.775, *SE* = 0.244, *df* = 160.49, *p* < .001), while the quadratic component was not significant (*b* = 0.094, *SE* = 0.245, *df* = 159.87, *p* = .701). There was no main effect of group (*b* = -0.017, *SE* = 0.066, *df* = 45.04, *p* = .792). However, the group x time (linear) interaction was significant (*b* = -0.554, *SE* = 0.244, *df* = 160.49, *p* = .025), indicating different decline slopes by condition; the group x time (quadratic) interaction was not significant (*b* = -0.203, *SE* = 0.245, *df* = 159.87, *p* = .409). Within-group simple slopes indicated significant linear declines in both conditions: Inclusion (*b* = -0.104, *SE* = 0.016, *df* = 161, *p* < .001) and Exclusion (*b* = -0.056, *SE* = 0.016, *df* = 160, *p* = .001). In sum, cortisol decreased across the session in both conditions, but exclusion flattened the decline.

For heart rate, there was a main effect of time (*F*(1, 42.42) = 7.81, *p* = .008) but no group x time interaction (*F*(1, 42.42) = 0.30, *p* = .587). Heart rate decreased slightly from baseline to during ostracism by -2.61 bpm (95% CI -4.49 to -0.73), irrespective of condition (Fig. 3B). For heart rate variability (RMSSD), there was a significant group x time interaction (*F*(1, 45.84) = 11.44, *p* = .001; Fig. 3C): exclusion reduced RMSSD from baseline (simple slope: -5.24 ms, 95% CI -9.61 to -0.87; *p* = .020), whereas inclusion showed the opposite pattern, with an increase from baseline (+ 5.04 ms, 95% CI 0.75 to 9.32; *p* = .022).

Perceived stress and tension showed no evidence of a group x time interaction from baseline to post-ostracism (stress: *F*(1, 48) = 1.28, *p* = .264; tension: *F*(1, 48) = 0.11, *p* = .738). NSSI urges likewise showed no clear interaction (*F*(1, 48) = 3.07, *p* = .086). The mean score on NSSI urges was 24.66 (SD = 28.51) on a 0–100 scale at baseline, and mean levels remained similar after the task across conditions. Thus, neither condition showed increases in subjective stress, tension, or NSSI urges (Figs. 3D–F).

## Discussion

The present study investigated multilevel stress responses to social rejection in adolescents who engage in NSSI using a novel, ecologically valid live ostracism paradigm (an adaptation of the Cyberball game [30]). Contrary to expectations, induced ostracism did not increase self-reported stress, tension, or NSSI urges relative to inclusion, yet clear physiological signatures of stress emerged. Specifically, ostracism was associated with an immediate reduction in heart rate variability (i.e., RMSSD) and a blunted decline in salivary cortisol over time, suggesting parasympathetic withdrawal coupled with attenuated HPA axis downregulation. These findings extend prior work on social threat sensitivity in NSSI [58–60] by demonstrating a decoupling between subjective and physiological responses to real-life exclusion. While affective states indicated limited conscious distress, autonomic and endocrine indicators pointed to covert physiological engagement, underscoring the value of multilevel stress response assessments in this population.

Regarding our first hypothesis (the HPA axis responses), we expected condition differences in salivary cortisol trajectories. However, our ostracism paradigm did not elicit a strong cortisol reactivity pattern in either group. Instead, exclusion attenuated the otherwise expected post-task decline, yielding a flatter downward slope relative to inclusion. This is consistent with blunted phasic reactivity and prolonged or less efficient recovery under social threat in adolescents with NSSI, a profile that converges with prior evidence for damped HPA responses in this population [10]. An alternative, complementary reading emphasizes social buffering: inclusion likely functioned as a safety signal, permitting a smoother decline, whereas exclusion removed this buffer and stalled deactivation (see [61]), even in the absence of a canonical cortisol spike.

Our second hypothesis addressed the impact of ostracism on autonomic regulation. Notably, parasympathetically governed heart rate variability decreased significantly during ostracism, indicating vagal withdrawal in response to social exclusion. This finding suggests that ostracism was experienced as a physiologically salient social threat and elicited a stress-related shift in autonomic regulation at the parasympathetic level. As lower heart rate variability has been associated with difficulties in emotion regulation and impaired impulse control [25, 26], this finding may be particularly relevant for understanding NSSI, as it points to a potential physiological pathway through which ostracism may increase vulnerability to self-injurious episodes. Our pattern of findings converges with that reported by Latina et al. [31], who likewise observed decreased heart rate variability during ongoing ostracism in a transdiagnostic adolescent sample. By contrast, we did not find a detectable effect of exclusion on heart rate. This may indicate that heart rate was less sensitive to the present manipulation, particularly given the relatively low physical demands of the seated, table-based task and the absence of the stronger activation typically observed in more intense social-evaluative stress paradigms such as the Trier Social Stress Test [48] or in physical challenge tasks (see [62]). Importantly, the absence of a heart rate effect does not diminish the significance of the RMSSD finding, which provides clear evidence of parasympathetic stress responding during ostracism.

Regarding our third hypothesis, which predicted that ostracism would increase subjective and affective responses including higher stress, tension, and NSSI urges, we found no impact of ostracism on any of these outcomes. This pattern is consistent with other Cyberball studies in clinical samples that likewise reported no immediate or prolonged affective responses, often attributed to the artificial, non-social context of traditional computerized tasks [31, 63]. Although we sought to address this limitation by implementing social rejection live and in person, ostracism in the present study was not accompanied by increases in self-reported stress or NSSI urges. This is notable because ostracism has been linked to subsequent NSSI engagement [58, 64]. However, in our data ostracism did not affect NSSI urges, diverging from daily-life findings [15, 19]. Importantly, this absence of subjective change occurred despite relatively clear physiological stress responses, pointing to a possible decoupling between subjective and psychobiological stress systems. Several explanations are conceivable, including limited interoceptive awareness in adolescents or internalization or overcompensation of perceived exclusion. In sum, Hypothesis 3 was not supported: social rejection did not alter stress, tension, or NSSI urges.

At the multisystem level, adolescents with NSSI perceived exclusion as threatening to basic psychological needs, yet subjective stress and NSSI urges did not change acutely at that stage. In contrast, autonomic withdrawal emerged rapidly, and cortisol subsequently declined more slowly across the session. This pattern suggests partial, lagged coupling between the ANS and the HPA axis, such that fast parasympathetic withdrawal is followed by a relatively blunted downregulation of HPA activity (a slower decline) rather than a discrete peak. Framed within the neurovisceral integration model [41, 42], the observed RMSSD reduction is consistent with vagal withdrawal during social exclusion. Although this pattern may be relevant to self-regulation processes implicated in NSSI, our design does not allow conclusions about poorer baseline regulatory capacity or heightened reactivity relative to adolescents without NSSI, as this would require a non-NSSI control group. More cautiously, the present findings are compatible with models linking vagal regulation to prefrontal control of emotion and stress responding. Indeed, neuroimaging studies have implicated the medial and ventrolateral prefrontal regions in processing social exclusion as well as regulating emotions in these contexts [39, 40, 65]. As these regions are thought to exert top-down inhibition of limbic threat systems, this framework may help contextualize our pattern of early vagal withdrawal and slower cortisol decline. Put differently, the absence of safety signals during exclusion may threaten social identity, reduce heart rate variability, and modestly impair endocrine regulation, thereby pushing adolescents toward states in which regulatory control is strained and impulsive coping becomes more likely (see [43]).

Our study is not without limitations. The live-interaction ostracism task effectively elicited exclusion and negative affect, but it has not yet been formally validated against the canonical computerized Cyberball [30], which limits direct comparability. Although we included an inclusion condition—standard in Cyberball research (see [47])—adding a nonclinical, age- and sex-matched comparison group would have strengthened inference. RMSSD is a widely used index of vagal activity, yet it can be influenced by biological sex, body mass index, medication use, respiration, posture, and motor activity [55], which could not be fully controlled for statistically in the present study. Physiological inferences should therefore be drawn with appropriate caution. Clinical characteristics typical of this population, particularly regarding comorbidities, may affect autonomic and endocrine measures. Our sample size limited the extent of covariate adjustment, although randomization likely reduced systematic bias. Finally, participants were adolescents (predominantly women and gender-diverse, many in treatment) so generalizability to men, non–treatment-seeking youth, young adults, and community samples is limited. The present study establishes a novel paradigm that future research can substantiate and refine in larger, more clinically and demographically stratified samples. Such research would also advance personalized medicine approaches, in which multilevel stress signatures could be used to identify adolescents requiring closer monitoring and to inform biological phenotyping of NSSI risk (see [66]).

### Conclusions and outlook

In conclusion, we found modest evidence for parasympathetic withdrawal and attenuated salivary cortisol downregulation in adolescents who engage in NSSI. These findings extend prior work by demonstrating that both autonomic and endocrine systems are activated by everyday social stressors and may contribute to NSSI vulnerability [10, 15, 67]. Although our paradigm was less potent than anticipated, it appears feasible and informative for studying ostracism in vivo, pending replication and formal validation. Clinically, the data underscore that stress systems do respond to social threat, supporting interventions that target autonomic regulation—for example, paced breathing, relaxation training, mindfulness exercises, and heart rate variability biofeedback [68, 69]. Recent work by Simundic et al. [29] suggests that even a brief mindfulness induction can buffer subsequent ostracism effects in adolescents with NSSI. At a broader level, school-based inclusion initiatives, social-media literacy programs, and anti-bullying efforts may bolster safety signals and reduce exposure to rejection-related contexts. Future research should probe mechanisms of post-stress regulation (e.g., perseverative cognition, social buffering) to refine targets that can help adolescents and young adults with NSSI, as well as individuals with emotion-regulation difficulties more generally.

## Data Availability

The datasets used and/or analyzed during the current study are available from the corresponding author on reasonable request.

## References

[1] International Society for the Study of Self-Injury. About self-Injury. 2025. Accessed 1 December 2025. https://www.itriples.org/aboutnssi

[2] Lloyd-Richardson EE, Perrine N, Dierker L, Kelley ML. Characteristics and functions of non-suicidal self-injury in a community sample of adolescents. Psychol Med. 2007;37(8):1183–92. 10.1017/S003329170700027X. 17349105 10.1017/S003329170700027X PMC2538378

[3] American Psychiatric Association. Diagnostic and statistical manual of mental disorders. 5th ed., text rev.; DSM-5-TR. 5th ed. Washington, DC: American Psychiatric Association. 2022. 10.1176/appi.books.9780890425787.

[4] Zetterqvist M. The DSM-5 diagnosis of nonsuicidal self-injury disorder: a review of the empirical literature. Child Adolesc Psychiatry Mental Health. 2015;9(1). 10.1186/s13034-015-0062-7. 10.1186/s13034-015-0062-7 PMC4584484 26417387

[5] Farkas BF, Takacs ZK, Kollárovics N, Balázs J. The prevalence of self-injury in adolescence: a systematic review and meta-analysis. Eur Child Adolesc Psychiatry. 2023;33:3439–58. 10.1007/s00787-023-02264-y. 37486387 10.1007/s00787-023-02264-y PMC11564408

[6] Xiao Q, Song X, Huang L, Hou D, Huang X. Global prevalence and characteristics of non-suicidal self-injury between 2010 and 2021 among a non-clinical sample of adolescents: A meta-analysis. Front Psychiatry. 2022;13:912441. 10.3389/fpsyt.2022.912441. 36032224 10.3389/fpsyt.2022.912441 PMC9399519

[7] Ferreira DBB, Santos RMS, Machado MCL, Rezende VHM, De Marco PG, Romano-Silva MA, et al. Suicidality and self-harm in adolescents before and after the COVID-19 pandemic: a systematic review. Front Psychiatry. 2025;16:1643145. 10.3389/fpsyt.2025.1643145. 41048920 10.3389/fpsyt.2025.1643145 PMC12491249

[8] Castellví P, Lucas-Romero E, Miranda-Mendizábal A, Parés-Badell O, Almenara J, Alonso I, et al. Longitudinal association between self-injurious thoughts and behaviors and suicidal behavior in adolescents and young adults: A systematic review with meta-analysis. J Affect Disord. 2017;215:37–48. 10.1016/j.jad.2017.03.035. 28315579 10.1016/j.jad.2017.03.035

[9] Griep SK, MacKinnon DF. Does Nonsuicidal Self-Injury Predict Later Suicidal Attempts? A Review of Studies. Archives Suicide Res. 2022;26(2):428–46. 10.1080/13811118.2020.1822244. 10.1080/13811118.2020.1822244 32985383

[10] Goreis A, Prillinger K, Bedus C, Lipp R, Mayer A, Nater UM, et al. Physiological stress reactivity and self-harm: A meta-analysis. Psychoneuroendocrinology. 2023;158:106406. 10.1016/j.psyneuen.2023.106406. 37783020 10.1016/j.psyneuen.2023.106406

[11] Kleindienst N, Bohus M, Ludäscher P, Limberger MF, Kuenkele K, Ebner-Priemer UW, et al. Motives for nonsuicidal self-injury among women with borderline personality disorder. J Nerv Mental Disease. 2008;196(3):230–6. 10.1097/NMD.0b013e3181663026 . PubMed PMID: 18340259. 10.1097/NMD.0b013e3181663026 18340259

[12] Bentley KH, Nock MK, Barlow DH. The four-function model of nonsuicidal self-injury: Key directions for future research. Clin Psychol Sci. 2014;2(5):638–56. 10.1177/2167702613514563.

[13] Nock MK, Prinstein MJ. A functional approach to the assessment of self-mutilative behavior. J Consult Clin Psychol. 2004;72(5):885–90. 10.1037/0022-006X.72.5.885. 15482046 10.1037/0022-006X.72.5.885

[14] Taylor PJ, Jomar K, Dhingra K, Forrester R, Shahmalak U, Dickson JM. A meta-analysis of the prevalence of different functions of non-suicidal self-injury. J Affect Disord. 2018;227:759–69. 10.1016/j.jad.2017.11.073. 29689691 10.1016/j.jad.2017.11.073

[15] Goreis A, Chang D, Klinger D, Zesch HE, Pfeffer B, Oehlke SM, et al. Impact of social media on triggering nonsuicidal self-injury in adolescents: a comparative ambulatory assessment study. bord personal disord emot dysregul. 2025;12(1):4. 10.1186/s40479-025-00280-9. 10.1186/s40479-025-00280-9 PMC11786379 39891274

[16] Janssens JJ, Kiekens G, Jaeken M, Kirtley OJ. A systematic review of interpersonal processes and their measurement within experience sampling studies of self-injurious thoughts and behaviours. Clin Psychol Rev. 2024;113:102467. 10.1016/j.cpr.2024.102467. 39084142 10.1016/j.cpr.2024.102467

[17] Peel-Wainwright K, Hartley S, Boland A, Rocca E, Langer S, Taylor PJ. The interpersonal processes of non‐suicidal self‐injury: A systematic review and meta‐synthesis. Psychol Psychother. 2021;94(4):1059–82. 10.1111/papt.12352. 34090311 10.1111/papt.12352

[18] Muehlenkamp J, Brausch A, Quigley K, Whitlock J. Interpersonal Features and Functions of Nonsuicidal Self-injury. Suicide Life Threat Behav. 2013;43(1):67–80. 10.1111/j.1943-278X.2012.00128.x. 23082783 10.1111/j.1943-278X.2012.00128.x

[19] Hepp J, Carpenter RW, Störkel LM, Schmitz SE, Schmahl C, Niedtfeld I. A systematic review of daily life studies on non-suicidal self-injury based on the four-function model. Clin Psychol Rev. 2020;82:1–21. 10.1016/j.cpr.2020.101888. 10.1016/j.cpr.2020.101888 PMC7680364 32949907

[20] Kothgassner OD, Robinson K, Goreis A, Ougrin D, Plener PL. Does treatment method matter? A meta-analysis of the past 20 years of research on therapeutic interventions for self-harm and suicidal ideation in adolescents. Borderline Personality Disorder Emot Dysregulation. 2020;7(1):9. 10.1186/s40479-020-00123-9. 10.1186/s40479-020-00123-9 PMC7216729 32426138

[21] Kothgassner OD, Goreis A, Robinson K, Huscsava MM, Schmahl C, Plener PL. Efficacy of dialectical behavior therapy for adolescent self-harm and suicidal ideation: a systematic review and meta-analysis. Psychol Med. 2021;51(7):1057–67. 10.1017/S0033291721001355. 33875025 10.1017/S0033291721001355 PMC8188531

[22] Kaess M, Hooley JM, Klimes-Dougan B, Koenig J, Plener PL, Reichl C, et al. Advancing a temporal framework for understanding the biology of nonsuicidal self- injury: An expert review. Neurosci Biobehav Rev. 2021;130:228–39. .022 PubMed PMID: 34450182. 34450182 10.1016/j.neubiorev.2021.08.022 PMC8783544

[23] Charmandari E, Tsigos C, Chrousos G. Endocrinology of the stress response. Annu Rev Physiol. 2005;67:259–84. 10.1146/annurev.physiol.67.040403. .120816 PubMed PMID: 15709959. 15709959 10.1146/annurev.physiol.67.040403.120816

[24] van Hoorn AC. Could affect regulation via vagal nerve self- stimulation be a maintaining factor in non-suicidal self-harm? Med Hypotheses. 2020;136:109498. 10.1016/j.mehy.2019.109498. 31759305 10.1016/j.mehy.2019.109498

[25] Beauchaine TP. Respiratory sinus arrhythmia: a transdiagnostic biomarker of emotion dysregulation and psychopathology. Curr Opin Psychol. 2015;3:43–7. 10.1016/j.copsyc.2015.01.017. 25866835 10.1016/j.copsyc.2015.01.017 PMC4389219

[26] Beauchaine TP, Thayer JF. Heart rate variability as a transdiagnostic biomarker of psychopathology. Int J Psychophysiol. 2015;98(2):338–50. 10.1016/j.ijpsycho.2015.08.004. 26272488 10.1016/j.ijpsycho.2015.08.004

[27] Kirschbaum C, Hellhammer DH. Salivary cortisol in psychoneuroendocrine research: Recent developments and applications. Psychoneuroendocrinology. 1994;19(4):313–33. 10.1016/0306-4530(94)90013-2. 8047637 10.1016/0306-4530(94)90013-2

[28] Sebastian C, Viding E, Williams KD, Blakemore SJ. Social brain development and the affective consequences of ostracism in adolescence. Brain Cogn. 2010;72(1):134–45. 10.1016/j.bandc.2009.06.008. 19628323 10.1016/j.bandc.2009.06.008

[29] Simundic A, Petrovic J, Khoury B, Geoffroy MC, Heath N. Evaluating a mindfulness induction for coping with social exclusion in emerging adults who engage in non-suicidal self-injury. Psychol Rep. 2025;00332941251383508. 10.1177/00332941251383508. 10.1177/00332941251383508 41005331

[30] Williams KD, Cheung CKT, Choi W, Cyberostracism. Effects of being ignored over the Internet. J Personal Soc Psychol. 2000;79(5):748–62. 10.1037/0022-3514.79.5.748. 10.1037//0022-3514.79.5.748 11079239

[31] Latina D, Goreis A, Sajko P, Kothgassner OD. Does Being Ignored on WhatsApp Hurt? A Pilot Study on the Effect of a Newly Developed Ostracism Task for Adolescents. J Clin Med. 2023;12(5):2056. 10.3390/jcm12052056. 36902843 10.3390/jcm12052056 PMC10004513

[32] Nezlek JB, Wesselmann ED, Wheeler L, Williams KD. Ostracism in everyday life. Group dynamics: theory, research, and practice. 2012;16(2):91–104. 10.1037/a0028029.

[33] Van Geel M, Goemans A, Vedder P. A meta-analysis on the relation between peer victimization and adolescent non-suicidal self-injury. Psychiatry Res. 2015;230(2):364–8. 10.1016/j.psychres.2015.09.017. 26391651 10.1016/j.psychres.2015.09.017

[34] Ariani TA, Putri AR, Firdausi FA, Aini N. Global prevalence and psychological impact of bullying among children and adolescents: a meta-analysis. J Affect Disord. 2025;385:119446. 10.1016/j.jad.2025.119446. 40393548 10.1016/j.jad.2025.119446

[35] Wilson E, Crudgington H, Morgan C, Hirsch C, Prina M, Gayer-Anderson C. The longitudinal course of childhood bullying victimization and associations with self‐injurious thoughts and behaviors in children and young people: A systematic review of the literature. J Adolesc. 2023;95(1):5–33. 10.1002/jad.12097. 36210652 10.1002/jad.12097 PMC10092090

[36] Williams KD, Ostracism. A temporal need-threat model. Adv Exp Soc Psychol. 2009;41:275–314.

[37] Groschwitz RC, Plener PL, Groen G, Bonenberger M, Abler B. Differential neural processing of social exclusion in adolescents with non-suicidal self-injury: An fMRI study. Psychiatry Research: Neuroimaging. 2016;255:43–9. 10.1016/j.pscychresns.2016.08.001. 27521517 10.1016/j.pscychresns.2016.08.001

[38] Perini I, Gustafsson PA, Hamilton JP, Kämpe R, Mayo LM, Heilig M, et al. Brain-based Classification of Negative Social Bias in Adolescents With Nonsuicidal Self-injury: Findings From Simulated Online Social Interaction. EClinicalMedicine. 2019;13:81–90. 10.1016/j.eclinm.2019.06.016. 31517265 10.1016/j.eclinm.2019.06.016 PMC6733998

[39] Domsalla M, Koppe G, Niedtfeld I, Vollstädt-Klein S, Schmahl C, Bohus M, et al. Cerebral processing of social rejection in patients with borderline personality disorder. Soc Cogn Affect Neurosci. 2014;9(11):1789–97. 10.1093/scan/nst176. 24273076 10.1093/scan/nst176 PMC4221221

[40] Eisenberger NI, Inagaki TK, Rameson LT, Mashal NM, Irwin R. An fMRI study of cytokine-induced depressed mood and social pain: the role of sex differences. 2010. 10.1016/j.neuroimage.2009.04.040 PMC2733873 19376240

[41] Thayer JF, Lane RD. A model of neurovisceral integration in emotion regulation and dysregulation. J Affect Disord. 2000;61(3):201–16. 10.1016/S0165-0327(00)00338-4. 11163422 10.1016/s0165-0327(00)00338-4

[42] Thayer JF, Hansen AL, Saus-Rose E, Johnsen BH. Heart Rate Variability, Prefrontal Neural Function, and Cognitive Performance: The Neurovisceral Integration Perspective on Self-regulation, Adaptation, and Health. ann behav med. 2009;37(2):141–53. 10.1007/s12160-009-9101-z. 19424767 10.1007/s12160-009-9101-z

[43] Höper S, Kaess M, Koenig J. Prefrontal cortex oxygenation and autonomic nervous system activity under transcutaneous auricular vagus nerve stimulation in adolescents. Auton Neurosci. 2022;241:103008. 10.1016/j.autneu.2022.103008. 35724559 10.1016/j.autneu.2022.103008

[44] Jankowski MS, Legasse AJ, Marques V, Delcourt ML, Haigh EAP. The smallest things make me emotional! Emotion reactivity in non-suicidal self-injury: trait, state, and physiological differences. Front Psychol. 2024;15:1309187. 10.3389/fpsyg.2024.1309187. 39246311 10.3389/fpsyg.2024.1309187 PMC11378346

[45] Parsons TD. Virtual reality for enhanced ecological validity and experimental control in the clinical, affective and social neurosciences. Front Hum Neurosci. 2015;9. 10.3389/fnhum.2015.00660. 10.3389/fnhum.2015.00660 PMC4675850 26696869

[46] Faul F, Erdfelder E, Lang AG, Buchner A. G*Power 3: A flexible statistical power analysis program for the social, behavioral, and biomedical sciences. Behav Res Methods. 2007;39(2):175–91. 10.3758/bf03193146. 17695343 10.3758/bf03193146

[47] Hartgerink CHJ, Van Beest I, Wicherts JM, Williams KD. The ordinal effects of ostracism: a meta-analysis of 120 cyberball studies. PLoS ONE. 2015;10(5). 10.1371/journal.pone.0127002. 10.1371/journal.pone.0127002 PMC4449005 26023925

[48] Kirschbaum C, Pirke KM, Hellhammer DH. The ‘Trier Social Stress Test’ – A Tool for Investigating Psychobiological Stress Responses in a Laboratory Setting. Neuropsychobiology. 1993;28(1–2):76–81. 10.1159/000119004. 8255414 10.1159/000119004

[49] Goreis A, Lozar A, List R, Pfeffer B, Prillinger K, Klinger D et al. Social exclusion alters attention and autonomic regulation in adolescents with nonsuicidal self-injury. PsyArXiv; 2025. https://osf.io/preprints/psyarxiv/384wk_v110.31234/osf.io/384wk_v1. 10.1038/s41398-026-04136-w PMC13376568 42177212

[50] Fox KR, Harris JA, Wang SB, Millner AJ, Deming CA, Nock MK. Self-Injurious Thoughts and Behaviors Interview—Revised: Development, reliability, and validity. Psychol Assess. 2020;32(7):677–89. 10.1037/pas0000819. 32324021 10.1037/pas0000819

[51] Kühner C, Bürger C, Keller F, Hautzinger M. Reliabilität und validität des revidierten Beck- Depressionsinventars (BDI-II). Befunde aus deutschsprachigen stichproben. Nervenarzt. 2007;78(6):651–6. 10.1007/s00115-006-2098-7 . PubMed PMID: 16832698. 16832698 10.1007/s00115-006-2098-7

[52] Beck AT, Steer RA, Brown GK. BDI-II: Beck depression inventory. San Antonio, TX: Pearson; 1996.

[53] Cohen S, Karmarck T, Mermelstein R. Perceived stress scale. Measuring stress: a guide for health and social scientists. 1994;10(2):1–2.

[54] Bohus M, Kleindienst N, Limberger MF, Stieglitz RD, Domsalla M, Chapman AL, et al. The Short Version of the Borderline Symptom List (BSL-23): Development and Initial Data on Psychometric Properties. Psychopathology. 2009;42(1):32–9. 10.1159/000173701. 19023232 10.1159/000173701

[55] Malik M, John Camm A, Thomas Bigger J, Breithardt G, Cerutti S, Cohen RJ, et al. Heart rate variability: Standards of measurement, physiological interpretation, and clinical use. Circulation. 1996;93(5):1043–65. 10.1161/01.cir.93.5.1043. 8598068

[56] Cohen J. Statistical power analysis for the behavioral sciences. Hillsdale, NJ: Routledge; 1988.

[57] Miller R, Plessow F, Rauh M, Gröschl M, Kirschbaum C. Comparison of salivary cortisol as measured by different immunoassays and tandem mass spectrometry. Psychoneuroendocrinology. 2013;38(1):50–7. 10.1016/j.psyneuen.2012.04.019. 22641005 10.1016/j.psyneuen.2012.04.019

[58] Cawley R, Pontin EE, Touhey J, Sheehy K, Taylor PJ. What is the relationship between rejection and self-harm or suicidality in adulthood? J Affect Disord. 2019;242:123–34. 10.1016/j.jad.2018.08.082. 30173060 10.1016/j.jad.2018.08.082

[59] Cummings LR, Mattfeld AT, Pettit JW, McMakin DL. Viewing Nonsuicidal Self-Injury in Adolescence Through a Developmental Neuroscience Lens: The Impact of Neural Sensitivity to Socioaffective Pain and Reward. Clin Psychol Sci. 2021;9(5):767–90. 10.1177/2167702621989323.

[60] Zhao J, Li A, Li K, Zhao F. How Is Rejection Sensitivity Linked to Non-Suicidal Self-Injury? Exploring Social Anxiety and Regulatory Emotional Self-Efficacy as Explanatory Processes in a Longitudinal Study of Chinese Adolescents. Behav Sci. 2024;14(10):943. 10.3390/bs14100943. 39457815 10.3390/bs14100943 PMC11505057

[61] Hay DE, Bleicher S, Azoulay R, Kivity Y, Gilboa-Schechtman E. Affective and cognitive impact of social overinclusion: a meta-analytic review of cyberball studies. Cogn Emot. 2023;37(3):412–29. 10.1080/02699931.2022.2163619. 36622872 10.1080/02699931.2022.2163619

[62] Skoluda N, Strahler J, Schlotz W, Niederberger L, Marques S, Fischer S, et al. Intra-individual psychological and physiological responses to acute laboratory stressors of different intensity. Psychoneuroendocrinology. 2015;51:227–36. 10.1016/j.psyneuen.2014.10.002. 25462896 10.1016/j.psyneuen.2014.10.002

[63] Iffland B, Sansen LM, Catani C, Neuner F. The trauma of peer abuse: effects of relational peer victimization and social anxiety disorder on physiological and affective reactions to social exclusion. Front Psychiatry. 2014;5. 10.3389/fpsyt.2014.00026. 10.3389/fpsyt.2014.00026 PMC3957367 24672491

[64] Song C, Liu L, Wang W. Distinguishing pathways from bullying victimization to nonsuicidal self-injury and to cyberaggression: Do perceived ostracism and depression mediate their links? Stress Health. 2024;40(3):e3337. 10.1002/smi.3337. 37876136 10.1002/smi.3337

[65] Brown RC, Plener PL, Groen G, Neff D, Bonenberger M, Abler B. Differential Neural Processing of Social Exclusion and Inclusion in Adolescents with Non-Suicidal Self-Injury and Young Adults with Borderline Personality Disorder. Front Psychiatry. 2017;8:267. 10.3389/fpsyt.2017.00267. 29238313 10.3389/fpsyt.2017.00267 PMC5712536

[66] Mürner-Lavanchy I, Koenig J, Reichl C, Josi J, Cavelti M, Kaess M. The quest for a biological phenotype of adolescent non-suicidal self-injury: a machine-learning approach. Transl Psychiatry. 2024;14(1):56. 10.1038/s41398-024-02776-4. 38267430 10.1038/s41398-024-02776-4 PMC10810046

[67] Goreis A, Pfeffer B, Hajek Gross C, Klinger D, Oehlke SM, Zesch H, et al. Attentional Biases and Nonsuicidal Self-Injury Urges in Adolescents. JAMA Netw Open. 2024;7(7):e2422892. 10.1001/jamanetworkopen.2024.22892. 39023890 10.1001/jamanetworkopen.2024.22892 PMC11258595

[68] Conrad A, Roth WT. Muscle relaxation therapy for anxiety disorders: It works but how? J Anxiety Disord. 2007;21(3):243–64. 10.1016/j.janxdis.2006.08.001. 16949248 10.1016/j.janxdis.2006.08.001

[69] Lehrer PM, Gevirtz R. Heart rate variability biofeedback: how and why does it work? Front Psychol. 2014;5. 10.3389/fpsyg.2014.00756. 10.3389/fpsyg.2014.00756 PMC4104929 25101026

